# The Role of NK Cells in the Control of Viral Infection in HTLV-1 Carriers

**DOI:** 10.1155/2019/6574828

**Published:** 2019-02-28

**Authors:** Camila F. Amorim, Natália B. Carvalho, José Abraão Neto, Silvane B. Santos, Maria Fernanda Rios Grassi, Lucas P. Carvalho, Edgar M. Carvalho

**Affiliations:** ^1^Serviço de Imunologia, Complexo Hospitalar Universitário Professor Edgard Santos, Universidade Federal da Bahia, Salvador, Bahia, Brazil; ^2^Departamento de Ciências Biológicas, Universidade Estadual de Feira de Santana, Feira de Santana, Bahia, Brazil; ^3^Laboratório de Pesquisas Clínicas do Instituto de Pesquisas Gonçalo Muniz-Fiocruz/Bahia, Brazil; ^4^Instituto Nacional de Ciência e Tecnologia de Doenças Tropicais (CNPq), Salvador, Bahia, Brazil

## Abstract

The cytotoxic activities of CD8^+^ T cells have been considered the main defense mechanism against the human T lymphotropic virus type 1 (HTLV-1). As with CD8^+^ T cells, NK cells can perform cytotoxic degranulation with production of cytotoxic mediators, such as perforins and granzymes. NK cells are also responsible for antibody-dependent cellular cytotoxicity (ADCC) against infected cells, but few studies have evaluated the role of NK cells in HTLV-1 infection. The aim of this study was to characterize the subsets and measure the frequency of NK cells in HTLV-1 carriers (HC) and in patients with HTLV-1-associated myelopathy/tropical spastic paraparesis (HAM/TSP) and correlate these findings with the proviral load and development of HAM/TSP. The diagnosis of HTLV-1 infection was performed with a detection antibody against viral antigens by ELISA and confirmed by Western blot. Phenotypic characterization of NK cells was performed by flow cytometry. The frequencies of CD56^+^, CD56^+^CD3^−^, CD56^+^CD16^+^, and CD56^dim^ cells were decreased in HAM/TSP patients. The frequency of CD56^+^CD3^−^ cells was inversely correlated with proviral load in HC but not in HAM/TSP patients. HAM/TSP patients showed decreased frequency of CD56^+^ and CD56^dim^ cells expressing CD16, the main receptor for ADCC. These data indicate that NK cells may play a key role in the control of HTLV-1 infection by preventing the progression of HC to HAM/TSP.

## 1. Introduction

The immune response against viral infection is based on effector mechanisms from both the innate and adaptive immune response. Among these mechanisms, the cytotoxicity mediated by NK cells and cytotoxic CD8^+^ T cells (CTL) is responsible for killing infected cells. In human T lymphotropic virus type 1 (HTLV-1) infection, while NK cells seek to limit the replication of the virus-infected cells and proviral load in the early stages of infection, the CTLs are responsible for the control of viral latency [1]. NK cells as well as CTLs have the ability to directly kill infected cells through the production of perforins and granzymes in cytotoxic granules. These granules are released from cytotoxic cells surrounded initially by a lipid bilayer containing lysosomal membrane glycoproteins, including CD107a. Granzymes induce programmed cell death (apoptosis) after invading the cytoplasm of the target cell through the pores formed in the cell membranes by perforins [2]. Additionally, NK cells have the ability to mediate antibody-dependent cellular cytotoxicity (ADCC) through the receptor CD16 by binding to antibodies opsonizing infected cells, leading to apoptosis [3]. Classical NK cells express NCAM-1 (CD56) on their membranes in high or low intensity may or may not express CD16 and lack CD3 expression [4]. Over the past 15 years, a “new” population of cells expressing both CD3 and CD56 and called NKT cells has been described [5]. Half of these cells express CD16 and all of them express classical T cell receptors (TCRs) that could recognize and respond to nonpeptide antigens like glycoproteins and polypeptides [5–8]. While NK cells have been mainly referred to as CD56^+^, CD56^+^CD3^−^, CD56^+^CD16^+^, CD56^dim^, and CD56^bright^, NKT cells are referred to as CD56^+^CD3^+^(CD16^+^/^−^).

In HTLV-1 infection, about 3% of infected subjects will develop HTLV-1-associated myelopathy/tropical spastic paraparesis (HAM/TSP) [9]. In such case, an invasion of infected and uninfected cells to the central nervous system (CNS) triggers an inflammatory, chronic, local response leading to nervous tissue damage. The Tax viral protein is responsible for increasing the expression of IL-2 receptor as well as gene expression related to the inflammatory response, resulting in a substantial lymphocyte activation, proliferation, and cytokine production by both CD4^+^ and CD8^+^ T cells [10]. The proviral load and production of inflammatory cytokines are increased in HAM/TSP patients compared to HTLV-1 carriers [11–13]. The immune response developed by cytotoxic cells in HTLV-1 is essential for controlling the proviral load, which may be critical in preventing the development of HAM/TSP. It is known that CTLs kill HTLV-1-infected cells through the recognition of the Tax protein, but the efficiency of this killing is impaired due to decreased expression of Tax and increased expression of another viral immunogenic gene, the HZB in HTLV-1-infected cells [14]. While the ligation of CD8^+^ T cells to cells expressing Tax is strong, these cells have an impaired ability to recognize HZB antigen. Moreover, there is a lack of studies evaluating the role of NK cells in HTLV-1. In this study, we phenotypically characterize NK and NKT cells in HTLV-1 infection, evaluate whether the expressions of CD16 and CD107a are altered, and correlate these findings with proviral load and development of HAM/TSP.

## 2. Methods

### 2.1. Ethical Statement

All HTLV-1-infected subjects were followed at the HTLV-1 clinic of the Complexo Hospitalar Universitário Professor Edgard Santos (COM-HUPES), Federal University of Bahia, Brazil. The study was approved by the Ethics Committee from the Federal University of Bahia, and all participants or patients were adults (>18 years old) and signed an informed consent.

### 2.2. Study Design and Case Definition

39 HTLV-1-infected subjects participated in this study, of which 20 were HTLV-1 carriers (HC) and 19 were diagnosed with HAM/TSP. 10 seronegative individuals (SN) not infected with HTLV-1 participated as controls. A pregnant woman, patients with other neurologic diseases not associated with HTLV-1, individuals coinfected with other pathogens, or patients on immunosuppressing drugs were excluded from this study. The diagnosis of HTLV-1 infection was established by ELISA (Murex HTLV-I+II, Abbot) and confirmed by Western blot (HTLV blot 2.4, Genelabs). Neurological involvement and motor dysfunction were determined by neurologic examination, evaluation of the expanded disability symptoms scale (EDSS), and determination of Osame's motor disability score (OMDS) [15]. Individuals with an OMDS and EDSS equal to 0 were considered HC. HAM/TSP diagnostic criteria were based on recommendations from an international consortium [15].

### 2.3. Isolation of Peripheral Blood Mononuclear Cells and Cell Cultures

Peripheral blood mononuclear cells (PBMCs) were obtained from heparinized blood of HTLV-1-infected subjects and SN and separated by density gradient with Ficoll-Hypaque (GE Healthcare Bio-Sciences). PBMCs from the interface were aspirated and washed with saline. These cells were resuspended in RPMI 1640 culture medium with L-glutamine and 25 mM HEPES (Gibco BRL), supplemented with 10% fetal bovine serum (FBS) and 0.5% gentamicin at 10 mg/mL (Gibco BRL). PBMCs were (a) stained with specific monoclonal antibodies for phenotypic analyses of NK and NKT cells by flow cytometry, (b) frozen in a solution of 10% DMSO, 50% FBS, and 40% RPMI for further proviral load quantification by PCR, and (c) cultured for 72 hours in the presence of anti-NKG2D monoclonal antibodies (R&D Systems, Minneapolis, Minnesota, USA).

### 2.4. Measuring Expression of Surface Markers in NK and NKT Cells to Characterize Cell Populations

**Figure 1:**
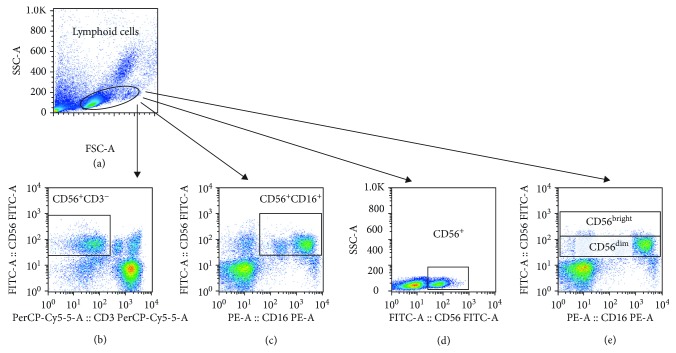
Strategy developed to characterize NK-like cells from HTLV-1-infected subjects and seronegative individuals. Peripheral blood mononuclear cells (PBMCs) were stained with monoclonal antibodies for characterization of NK-like cells by flow cytometry. Lymphoid cells were selected by size (SSC) and granularity (FSC) (a), and then cells were characterized by CD56^+^CD3^−^ (b), CD56^+^CD16^+^ (c), CD56^+^ cells (d), and CD56^dim^ and CD56^bright^ (e).

**Figure 2:**
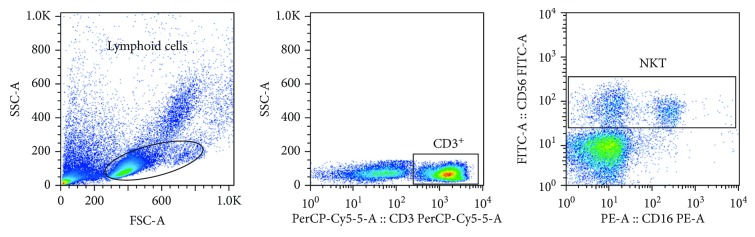
Strategy developed to characterize CD56^+^CD3^+^ NKT cells from HTLV-1-infected subjects and seronegative individuals. Peripheral blood mononuclear cells (PBMCs) were stained with monoclonal antibodies for characterization of CD56^+^CD3^+^ NKT cells by flow cytometry. Lymphoid cells were selected by size (SSC) and granularity (FSC) (A), and then NKT cells were gated from a CD3^+^ plot, classified as CD56^+^CD3^+^(CD16^+/-^).

The ex vivo frequency of NK and NKT cell populations and expression of CD16 and CD107a (a degranulation marker) were determined using PBMCs from HC, HAM/TSP patients, and SN. Cells were stained with monoclonal antibodies (anti-CD3, anti-CD56, anti-CD16, and anti-CD107a) (eBioscience or R&D Systems) for 20 minutes at 4°C. PBMCs were washed with PBS and then fixed with 2% paraformaldehyde. Cells were then analyzed on the FacsCanto II flow cytometer (BD Biosciences, San Jose, CA). Analyses were performed using FlowJo software version 10 (Tree Star, Ashland, OR). Lymphoid cells were selected by size (SSC) and granularity (FSC) and then characterized as CD56^+^CD3^−^, CD56^+^CD16^+^, and CD56^+^ cells were further divided as CD56^dim^ or CD56^bright^. These cells are herein called NK-like cells for having strong similarities with and core functions known to be associated with classical NK cells. To look specifically at NKT cells, we analyzed a subset of cells gated from lymphoid cells and then gated for CD3^+^ cells, classified as CD56^+^CD3^+^(CD16^+^/^−^). The gating strategy is shown in Figures 1 and 2. In this study, fluorescence minus one (FMO) was used as an internal experiment control for flow cytometry in the evaluation of CD107a expression, with the limit of fluorescence as a negative control.

### 2.5. DNA Extraction and Proviral Load

DNA extraction was performed by the salting-out method as described by Amorim et al. [16]. Briefly, PBMCs were thawed, washed with saline, and treated with a solution containing proteinase K buffer 5x, proteinase K, SDS, and 20% distilled H_2_O in order to disrupt and lyse the protein and lipid content of biological membranes and the cytoplasm. The cell solution was maintained at 37°C in the absence of CO_2_ for 24 hours for complete cell lysis. Thereafter, 6 M NaCl was added to the solution for the neutralization of DNA-charged phosphate groups and to induce folding. Then, the material was centrifuged at 13,000g for 10 minutes to separate the genetic content (supernatant) from the lysed cell content (pellet). 99.5% absolute ethanol was added to the DNA to dry and promote folding. Then, the DNA was transferred to a 70% ethanol solution for hydration. Distilled H_2_O was added to the DNA samples, which were then kept at -20°C until quantification of proviral load.

The determination of the proviral load was performed by TaqMan PCR (Applied Biosystems) as previously described by Dehée et al. [17]. Albumin DNA was used as an endogenous reference. Amplification and data acquisition were carried out using the ABI Prism 7700 Sequence detector system (Applied Biosystems). Standard curves were generated using a 10-fold serial dilution of a double-stranded plasmid (pcHTLV-ALB). All standard dilutions, control, and individual samples were run in duplicate for both HTLV-1 and albumin DNA quantification. The normalized value of the HTLV-1 proviral load was calculated as the ratio of (HTLV-1 DNA average copy number/albumin DNA average copy number) ×2 × 10^6^ and expressed as the number of HTLV-1 copies/10^6^ cells.

### 2.6. Impact of NKG2D Neutralization on the Frequency of NK-Like Cells Expressing CD107a and the Proviral Load

To evaluate the impact of NKG2D neutralization on the expression of CD107a and the proviral load, 3 × 10^6^ PBMCs from 7 HTLV-1-infected individuals were incubated (37°C, 5% of CO_2_) with media or in the presence of anti-NKG2D antibody (20 *μ*g/mL) for 3 days. The frequency of NK cells expressing CD107a was determined by FACS analysis, and the proviral load was determined by PCR.

### 2.7. Statistical Analyses

The nonparametric statistical tests were used in this study. The Mann-Whitney test was used to assess differences between the groups studied. The sum-rank test was used to compare data in the same experiment using different conditions. The Spearman *r* test was used to determine correlations. Data were expressed as median values (minimal to maximal values). GraphPad Prism 5 (San Diego, CA) was used for statistical evaluation, and a *P* < 0.05 was considered significant.

## 3. Results

The demographic features and proviral load from SN, HC, and patients with HAM/TSP are shown in Table 1. The HC and HAM/TSP patients were significantly older than SN, but there was no difference regarding age between the HTLV-1 positive groups. The median proviral load was significantly higher in HAM/TSP patients (72,438 copies/10^6^ cells) than in HC (16,190 copies/10^6^ cells) (Table 1).

### 3.1. Characterization of NK and NKT Cells from HTLV-1-Infected Subjects

The frequencies of NK and NKT cells were measured by flow cytometry using specific monoclonal antibodies against surface molecules that are classically to identify these cells. The individual cell populations were gated from a lymphoid cell population (Figure 1(a)) and them classified as CD56^+^, CD56^+^CD3^−^, CD56^+^CD16^+^, CD56^dim^, and CD56^bright^ (Figures 1(b)–1(e)). NKT cells were also selected from the lymphoid cells by size and cell granularity, gated as CD3^+^, and then characterized as CD56^+^CD3^+^ (Figure 2).

### 3.2. The Frequency of NK-Like Cells Was Decreased in HAM/TSP Patients and Inversely Correlated with Proviral Load in HC

Patients with HAM/TSP showed significant decreased frequencies of CD56^+^CD3^−^, CD56^+^CD16^+^, CD56^+^, and CD56^dim^ cells, with exception of CD56^bright^ cells and CD56^+^CD3^+^ NKT cells, compared to the frequencies observed in SN and HC (Table 2).

**Figure 3:**
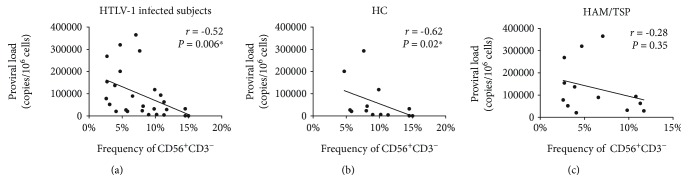
Correlation between proviral load and the frequency of CD56^+^CD3^−^ cells in individuals infected with HTLV-1. Correlation between proviral load and frequency of CD56^+^CD3^−^ in HTLV-1-infected subjects (HC+HAM/TSP) (a), in HC (b), and in HAM/TSP patients (c), separately. The Spearman test was used in statistical analyses ^∗^*P* < 0.05.

We also determined if the frequency of these cells was correlated with proviral load. There was a significant inverse correlation between the proviral load and the frequency of CD56^+^CD3^−^ cells in HTLV-1-infected subjects, combining all HC and HAM/TSP patients (*r* = -0.52 and *P* = 0.006) (Figure 3(a)). Moreover, when we performed the correlation taking into account CD56^+^CD3^−^ cells, there was a significant inverse correlation with the proviral load and the frequency of CD56^+^CD3^−^ cells in HC (*r* = -0.62 and *P* = 0.02) but not in HAM/TSP patients (*r* = -0.28 and *P* = 0.35) (Figures 3(b) and 3(c)). There was no significant correlation between proviral load and the frequency of CD56^+^CD16^+^, CD56^+^, or CD56^dim^ cells in both HC and patients with HAM/TSP.

### 3.3. The Frequency of CD56^+^ and CD56^dim^ Cells Expressing CD16 Is Decreased in HAM/TSP

**Figure 4:**
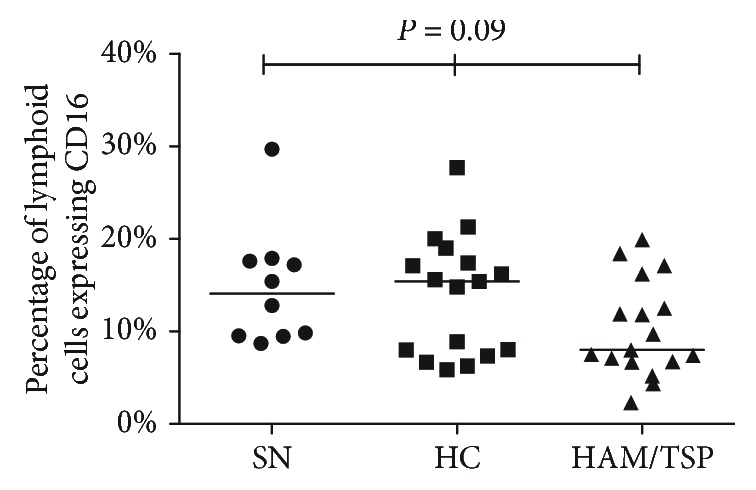
Frequency of lymphoid cells expressing CD16 in seronegative individuals and HTLV-1-infected subjects. Frequency of lymphoid cells expressing CD16 in HTLV-1 carriers, HAM/TSP patients, and seronegative individuals. The Kruskal-Wallis test was used in statistical analyses. SN: seronegative individuals; HC: HTLV-1 carriers.

To evaluate the expression of the main receptor involved with ADCC against infected cells, we assessed the frequency of cells expressing CD16. We observed that HAM/TSP patients showed a significantly decreased frequency of lymphoid cells expressing CD16 in general (8%, compared to HC, 15.1%, and SN, 14%), but we could not observe statistically significant differences (*P* = 0.09) (Figure 4).

**Figure 5:**
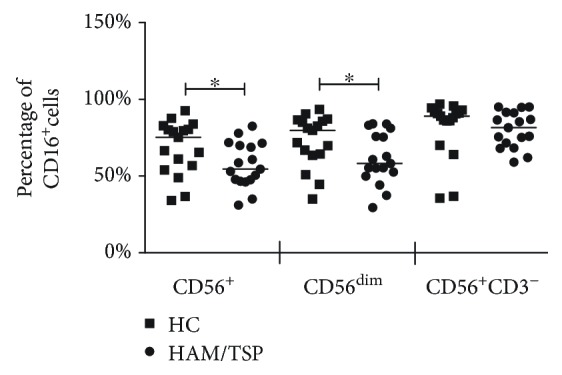
Decreased frequency of CD56^+^ and CD56^dim^ cells expressing CD16 in HAM/TSP patients. Frequency of CD56^+^, CD56^dim^, and CD56^+^CD3^−^ cells expressing CD16 in HTLV-1 carriers and HAM/TSP patients. The Mann-Whitney test was used in statistical analyses. ^∗^*P* < 0.05. HC: HTLV-1 carriers.

We also observed that the frequency of CD56^+^ cells (53.1%), specifically CD56^dim^ cells expressing CD16 (58.2%) from HAM/TSP patients, was decreased compared to HC, 75.3% and 79.8%, respectively (*P* < 0.05). CD56^+^CD3^−^ cells from HAM/TSP patients did not exhibit a decrease in the percentage of CD16^+^ cells in comparison to HC (Figure 5).

### 3.4. Expression of CD107a in NK Cells Is Increased in HTLV-1 Infection

CD107a is a classical surface marker for degranulation of cytotoxic mediators by cytotoxic cells. When the cell is activated, granules containing perforins and granzymes are released from cytotoxic cells through vacuoles expressing CD107a on the membrane.

We observed that the frequency of NK-like cells expressing CD107a was significantly higher in HC and HAM/TSP compared to SN (*P* < 0.05). We did not observe differences between the HC and HAM/TSP groups (Table 3).

In addition to increased cell frequencies, HTLV-1-infected subjects also presented significantly increased MFI (mean of fluorescent intensity) of CD107a expression by NK-like cell subsets compared to SN (*P* < 0.05). However, we did not observe significant differences between HC and HAM/TSP groups. Interestingly to note, the subset that presented the highest intensity of CD107a expression compared to the other subsets was the CD56^+^CD3^+^ NKT cells in SN, HC, and HAM/TSP groups (Table 3).

### 3.5. Impact of Blockage of NKG2D on the Degranulation of NK Cells and on Proviral Load

**Figure 6:**
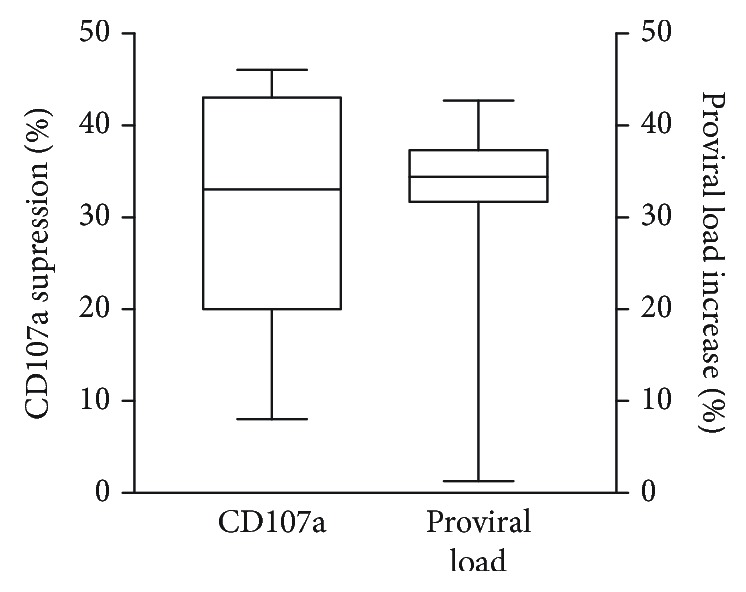
Impact of NKG2D neutralization on the frequency of CD56^+^CD3^−^ cells expressing CD107a and on the proviral load. PBMCs from 7 HTLV-1-infected individuals were incubated in the presence or absence of anti-NKG2D antibody (20 *μ*g/mL) for 3 days. The frequency of CD56^+^CD3^−^ cells expressing CD107a was determined by FACS. Data represents the median of the percentage of suppression of cells expressing CD107a and the percent increase in the proviral load after exogenous addition of anti-NKG2D (*P* < 0.05), Wilcoxon rank-sum test.

NKG2D is an activation marker on NK and CD8 T cells that leads to degranulation of cytotoxic cells and expression of CD107a. Our results showed that the frequency of CD56^+^CD3^−^ cells was inversely correlated with the proviral load. In order to determine if NK cell degranulation was associated with the decrease in proviral load, we blocked with a monoclonal antibody against NKG2D and measured the frequency of CD56^+^CD3^−^ cells expressing CD107a as well as the proviral load (Figure 6). While the blockage of NKG2D decreased the frequency of cells expressing CD107a by 36.3% (*P* < 0.05), there was an increase in proviral load on the order of 30.5% (*P* < 0.05).

## 4. Discussion

HAM/TSP is a chronic, progressive, neurological disease that leads to weakness of the inferior limbs, spasticity, and the inability to walk. A high HTLV-1 proviral load is the main risk factor for HAM/TSP [18], and the control of viral replication by CTLs is considered the main host defense mechanism against HTLV-1. NK cells also develop cytotoxic mechanisms that can kill infected cells in early infection stages. In human immunodeficiency virus type 1 (HIV-1), cytomegalovirus (CMV), and HTLV-1 infections, the viruses have the ability to downregulate HLA molecules in infected cells, impairing killing mediated by CD8^+^ T cells [19–22]. NK cells play an important role against viral infections because they can overcome this deficiency through their receptors (such as KIR, NKG2A, and ILT2) that will recognize the lack of MHC antigen presentation, starting the cascade of signaling, activation, and production of cytotoxic mediators [23–25]. In this study, we show that the frequency of NK-like cells was significantly decreased in HAM/TSP, especially in CD16^+^ cells, and there was an inverse correlation between the frequency of NK-like cells and proviral load in HC but not in HAM/TSP. Moreover, we showed that the blockage of NKG2D by a monoclonal antibody significantly increased the proviral load *in vitro*.

NK and NKT cells from humans are mainly characterized by the presence of CD56, as well as the presence or absence of CD16 and CD3. A previous study showed that the frequency of CD16^+^, CD11b^+^, CD56^+^, CD16^+^CD56^−^, CD56^+^CD16^+^, CD16^−^CD56^+^, CD16^+^CD8^−^, CD16^+^CD3^+^, and CD3^+^CD56^+^CD16^+^ was reduced in HAM/TSP [26, 27]. It was also noted that the frequency of CD16^+^CD3^+^ cells was inversely correlated with lymphocyte proliferation, and the frequency of NK cells and NKT cells was lower in HAM/TSP patients in comparison to HTLV-1 carriers [27]. Moreover, CD56^+^CD16^dim^ and CD56^+^CD16^−^ cells spontaneously proliferate in HTLV-1-infected subjects, and the rate of proliferation was correlated with the proviral load [28].

CD56^+^CD3^−^ cells are the classical NK cells with innate properties, and the possibility of including NKT cells in this gating strategy is excluded by the absence of CD3. In the present study, we observed that the frequency of CD56^+^CD3^−^ cells was decreased in HAM/TSP patients compared to HC, and there was a significant inverse correlation with proviral load in HC patients but not in HAM/TSP patients. These data initially suggested that decreased frequencies of cytotoxic NK cells in the peripheral blood of HAM/TSP patients might have an impact on how the host immune response controls retroviral proliferation.

Among CD56^+^ cells, 10% are CD56^bright^ cells, which are known for having critical importance in contributing to cytokine production in viral infections, specifically through the production of IL-1, IL-2, and IL-18 [29]. These cells do not seem to have a decrease in frequency in HAM/TSP patients; however, the frequency of CD56^dim^ cells is decreased in these patients compared to HC. CD56^dim^ cells constitute 90% of all the cells expressing CD56 and are known for having an important role in cytotoxicity because they express increased levels of CD16 and have lymphokine-activated killer (LAK) activity and natural killing ability against infected cells [29].

The receptor CD16 (Fc*γ*RIII) is responsible for binding to the Fc region of antibodies that opsonize infected cells and trigger apoptosis in these cells, destroying intracellular provirus through a process known as antibody-dependent cell-mediated cytotoxicity (ADCC). In the present study, in addition to a decreased frequency of CD56^dim^ cells in HAM/TSP, we also observed both a significant decrease in the frequency of NK CD56^+^CD16^+^ cells and a significant decrease in overall expression of CD16 in lymphoid cells in the peripheral blood of HAM/TSP patients compared to HC. These data together suggest that a decreased frequency of cells expressing CD16 to promote ADCC against HTLV-1-infected cells might also have a significant impact on proviral load control in HAM/TSP patients. No previous study has assessed ADCC in NK cells in HTLV-1 infection, and functional studies to evaluate ADCC in the context of HTLV-1 are necessary. The importance of CD16 in HIV-1 infection is well studied and points to the protective role of ADCC by NK cells in controlling viremia and disease progression [30]. Although it is possible that ADCC is impaired in patients with HAM/TSP, the reduced expression of CD16 may have other explanations. CD16 is cleaved very rapidly after binding to opsonizing antibodies to trigger apoptotic events [31–33]. Thus, we cannot rule out that activation of the CD16 receptor and subsequent cleavage might be occurring in HAM/TSP.

Regarding NKT cells, two studies have shown that the frequency of NKT cells (using anti-V*α*24, anti-V*β*11, and anti-CD1d as cell surface markers) was reduced in HAM/TSP [34, 35]. We did not confirm that the frequency was decreased in HTLV-1 infection. These data can be explained by the difference in the type of NKT cell surface staining that was used in the methodology of these papers. Other studies have mainly characterized NKT cells as V*α*24^+^, V*β*11^+^, CD1d^+^, CD16^+^CD3^+^, or CD56^+^CD16^dim^, and here, we characterized them as CD56^+^CD3^+^(CD16^+^/^−^). As the frequency of NKT cells in seronegative individuals, HTLV-1 carriers, and HAM/TSP was 6.4%, 4.8%, and 4.1%, respectively, in this study, we cannot rule out that a decreased frequency of these cells might be observed in HAM/TSP with an increase in the number of analyzed individuals or by changing the type of staining.

As NK cells are important antiviral components able to identify and kill infected targets, a decreased frequency of these cells may impair viral killing. The role of the frequency of NK cells in control of viral load is recognized in other infections. There is an inverse correlation between the frequency of NK cells and the levels of HIV-RNA in the plasma of patients with primary HIV infection [36]. Recently, it was described that fatal cases of Ebola infections presented lower frequencies of NK cells compared to nonfatal cases [37]. Additionally, NK cells from the liver of hepatitis C virus-infected patients are more activated than NK cells from the blood of the same individuals, and IFN-based therapy increases NK cell frequencies while decreasing viral load [38].

The intensity of CD107a expression is an indication that cells are promoting cytotoxicity. We observed that NK and NKT cells of HTLV-1-infected subjects expressed significantly more CD107a than did those of SN, but there were no differences between HC and HAM/TSP patients. Even though the ex vivo expression of CD107a by NK cells is similar between HC and HAM/TSP patients, we speculate that decreased frequencies of NK cells developing cytotoxicity in HAM/TSP patients might be one of the reasons for the impaired control of proviral proliferation.

HTLV-1 preferentially infects CD4 and CD8 T cells and also has the ability to stimulate exaggerated proliferation of these populations in the peripheral blood [39]. One of the possible explanations for the decreased frequency of NK cells is that the production of lymphocytes in lymphoid organs is polarized towards CD4 and CD8 T cells instead of other cell populations such as NK cells. Because in HAM/TSP patients the proviral load is increased compared to HC, there is more viral stimulation which is associated with this cell production polarization, leading to lower numbers of circulating NK cells.

It is possible that not only the frequency of NK cells is impaired HTLV-1 infection but also the functionality. In this case, it is also possible that HAM/TSP as well as HC might present cytotoxic defects in protecting the host against HTLV-1.

We recognize that one limitation of the present study is the lack of functional studies showing that HTLV-1-infected cells are killed by NK cells. However, our observation that in addition to the decrease in the frequency of NK-like cells in patients with HAM/TSP, the blockage of NKG2D also decreased the frequency of NK cells expressing CD107a and increased the proviral load, which argues in favor of NK cells killing HTLV-1-infected cells and participating in the control of the proviral load.

## 5. Conclusions

The decreased frequency of NK-like cells in patients with HAM/TSP, the documentation of an inverse correlation between the frequencies of these cells with proviral load in HC but not in HAM/TSP, and the documentation that *in vitro* blockage of NKG2D increases proviral load indicate that cytotoxicity mediated by NK cells may be an important mechanism in the control of proviral load and in preventing progression from HC status to HAM/TSP.

## Figures and Tables

**Table 1:** Demographic features and proviral load in HTLV-1 carriers and patients with HAM/TSP.

	Seronegative individuals (SN) (*n* = 10)	HTLV-1 carriers (HC) (*n* = 20)	HAM/TSP (*n* = 19)	*P* value
Age (years)	30 (23-45)	52 (31-69)	63 (36-76)	<0.0001^∗^^a,b^
Gender (M/F)	1/9	8/12	9/10	0.12^∗∗^
Proviral load (copies/10^6^cells)	Not applicable	16,190 (110-292,916)	72,438 (20,000-365,075)	<0.0001^∗∗∗^

^∗^Kruskal-Wallis test with Dunn's test (^a^HC × SN, ^b^HAM/TSP × HC); ^∗∗^chi-square (*χ*^2^); ^∗∗∗^Mann-Whitney test. Proviral load is represented by median (minimal and maximal values).

**Table 2:** NK and NKT cell frequencies in HTLV-1-infected subjects.

Frequency of cells (%)	Seronegative individuals (SN) (*n* = 10)	HTLV-1 carriers (HC) (*n* = 20)	HAM/TSP (*n* = 19)
CD56^+^CD3^−^	7.9 (4.5-15.3)	8.9 (2.8-15)	5.8 (1.9-11.7)^a,b^
CD56^+^CD16^+^	10.1 (5.3-16.2)	10.7 (3.2-22.9)	7.2 (1.8-15.8)^a,b^
CD56^+^	16.4 (11.6-23.8)	13.3 (6.4-26.4)	11.2 (4.6-19.4)^a,b^
CD56^dim^	12.9 (9-21.3)	12.2 (5-27.4)	9.5 (3.4-19.4)^a,b^
CD56^bright^	0.9 (0.5-3.4)	0.8 (0.07-1.5)	0.7 (0.2-1.3)
NKT	6.4 (3.5-11.5)	4.6 (2.2-14.7)	4.1 (1.9-9.7)

The Mann-Whitney test was used for statistical analyses. Data are represented by median (minimal and maximal values); ^a^SN × HAM/TSP (*P* < 0.05); ^b^HC × HAM/TSP (*P* < 0.05).

**Table 3:** Percentage of cells expressing CD107a and MFI of CD107a expression from HTLV-1-infected subjects.

	Seronegative individuals (SN) (*n* = 10)	HTLV-1 carriers (HC) (*n* = 15)	HAM/TSP (*n* = 16)
Percentage of cells expressing CD107a (minimal-maximal)			
CD56^+^CD3^−^	2.3 (0.8-12)	6.7 (1.4-41.9)^a^	6.9 (1.8-57.6)^b^
CD56^+^CD16^+^	2.8 (1.5-14)	6.5 (1.7-40)^a^	7.7 (3.4-70.8)^b^
CD56^+^	4.6 (2.6-12.4)	8 (2.2-39.2)^a^	11.1 (4.4-61.9)^b^
CD56^dim^	5.1 (3.2-12.9)	8.3 (2.4-40.1)^a^	11.8 (4.4-62)^b^
CD56^bright^	0.9 (0-11.7)	4.9 (0.7-30.8)^a^	5.4 (0-66.7)^b^
NKT	8.4 (6.6-11.6)	11.8 (6-52.7)^a^	15.8 (6.1-50)^b^
MFI (minimal-maximal)			
CD56^+^CD3^−^	13.1 (9.6-49.2)	22.6 (15.3-48.2)^a^	18.7 (16.3-37.2)^b^
CD56^+^CD16^+^	15.1 (10.2-19.7)	23.5 (1.7-92.1)^a^	23.8 (18.1-48.1)^b^
CD56^+^	17.6 (11.1-52)	27.1 (18.7-76)^a^	27 (21-47)^b^
CD56^dim^	18 (10.9-53.9)	28.9 (2.4-77.2)^a^	26.7 (2.4-50.1)^b^
CD56^bright^	15.5 (9.5-43.3)	24.4 (0.9-79.9)^a^	26.7 (17.1-38.3)^b^
NKT	25.9 (13.9-52.4)	34.4 (6-85)^a^	29.8 (16.7-75.7)

The Mann-Whitney test was used for statistical analyses. Data are represented by median (minimal and maximal values); ^a^SN × HC (*P* < 0.05); ^b^SN × HAM/TSP (*P* < 0.05).

## Data Availability

The data used to support the findings of this study are included within the article.

## References

[1] Bangham C. R. M. (2009). CTL quality and the control of human retroviral infections. *European Journal of Immunology*.

[2] Thiery J., Keefe D., Boulant S. (2011). Perforin pores in the endosomal membrane trigger the release of endocytosed granzyme B into the cytosol of target cells. *Nature Immunology*.

[3] Fast L. D., Hansen J. A., Newman W. (1981). Evidence for T cell nature and heterogeneity within natural killer (NK) and antibody-dependent cellular cytotoxicity (ADCC) effectors: a comparison with cytolytic T lymphocytes (CTL). *The Journal of Immunology*.

[4] Caligiuri M. A. (2008). Human natural killer cells. *Blood*.

[5] Bendelac A., Savage P. B., Teyton L. (2007). The biology of NKT cells. *Annual Review of Immunology*.

[6] Godfrey D. I., Pellicci D. G., Smyth M. J. (2004). The elusive NKT cell antigen--is the search over?. *Science*.

[7] Holderness J., Hedges J. F., Ramstead A., Jutila M. A. (2013). Comparative biology of *γδ* T cell function in humans, mice, and domestic animals. *Annual Review of Animal Biosciences*.

[8] Pellicci D. G., Clarke A. J., Patel O. (2011). Recognition of *β*-linked self glycolipids mediated by natural killer T cell antigen receptors. *Nature Immunology*.

[9] Gessain A., Vernant J. C., Maurs L. (1985). Antibodies to human T-lymphotropic virus type-I in patients with tropical spastic paraparesis. *The Lancet*.

[10] Osame M. (2002). Pathological mechanisms of human T-cell lymphotropic virus type I-associated myelopathy (HAM/TSP). *Journal of NeuroVirology*.

[11] Nagai M., Yamano Y., Brennan M. B., Mora C. A., Jacobson S. (2001). Increased HTLV-I proviral load and preferential expansion of HTLV-I Tax-specific CD8+ T cells in cerebrospinal fluid from patients with HAM/TSP. *Annals of Neurology*.

[12] Olindo S., Lézin A., Cabre P. (2005). HTLV-1 proviral load in peripheral blood mononuclear cells quantified in 100 HAM/TSP patients: a marker of disease progression. *Journal of the Neurological Sciences*.

[13] Santos S. B., Porto A. F., Muniz A. L. (2004). Exacerbated inflammatory cellular immune response characteristics of HAM/TSP is observed in a large proportion of HTLV-I asymptomatic carriers. *BMC Infectious Diseases*.

[14] MacNamara A., Rowan A., Hilburn S. (2010). HLA class I binding of HBZ determines outcome in HTLV-1 infection. *PLoS Pathogens*.

[15] De Castro-Costa C. M., Araújo A. Q. C., Barreto M. M. (2006). Proposal for diagnostic criteria of tropical spastic paraparesis/HTLV-I-associated myelopathy (TSP/HAM). *AIDS Research and Human Retroviruses*.

[16] Amorim C. F., Souza A. S., Diniz A. G., Carvalho N. B., Santos S. B., Carvalho E. M. (2014). Functional activity of monocytes and macrophages in HTLV-1 infected subjects. *PLoS Neglected Tropical Diseases*.

[17] Dehée A., Césaire R., Désiré N. (2002). Quantitation of HTLV-I proviral load by a TaqMan real-time PCR assay. *Journal of Virological Methods*.

[18] dos Santos Brito Silva Furtado M., Andrade R. G., Romanelli L. C. F. (2012). Monitoring the HTLV-1 proviral load in the peripheral blood of asymptomatic carriers and patients with HTLV-associated myelopathy/tropical spastic paraparesis from a Brazilian cohort: ROC curve analysis to establish the threshold for risk disease. *Journal of Medical Virology*.

[19] Ahn K., Angulo A., Ghazal P., Peterson P. A., Yang Y., Fruh K. (1996). Human cytomegalovirus inhibits antigen presentation by a sequential multistep process. *Proceedings of the National Academy of Sciences of the United States of America*.

[20] Scully E., Alter G. (2016). NK cells in HIV disease. *Current HIV/AIDS Reports*.

[21] Jeffery K. J. M., Siddiqui A. A., Bunce M. (2000). The influence of HLA class I alleles and heterozygosity on the outcome of human T cell lymphotropic virus type I infection. *The Journal of Immunology*.

[22] Jeffery K. J. M., Usuku K., Hall S. E. (1999). HLA alleles determine human T-lymphotropic virus-I (HTLV-I) proviral load and the risk of HTLV-I-associated myelopathy. *Proceedings of the National Academy of Sciences of the United States of America*.

[23] Braud V. M., Tomasec P., Wilkinson G. W. G. (2002). Viral evasion of natural killer cells during human cytomegalovirus infection. *Current Topics in Microbiology and Immunology*.

[24] Jost S., Altfeld M. (2012). Evasion from NK cell-mediated immune responses by HIV-1. *Microbes and Infection*.

[25] Kärre K. (2002). NK cells, MHC class I molecules and the missing self. *Scandinavian Journal of Immunology*.

[26] Wu X. M., Osoegawa M., Yamasaki K. (2000). Flow cytometric differentiation of Asian and Western types of multiple sclerosis, HTLV-1-associated myelopathy/tropical spastic paraparesis (HAM/TSP) and hyperIgEaemic myelitis by analyses of memory CD4 positive T cell subsets and NK cell subsets. *Journal of the Neurological Sciences*.

[27] Yu F., Itoyama Y., Fujihara K., Goto I. (1991). Natural killer (NK) cells in HTLV-I-associated myelopathy/tropical spastic paraparesis — decrease in NK cell subset populations and activity in HTLV-I seropositive individuals. *Journal of Neuroimmunology*.

[28] Norris P. J., Hirschkorn D. F., DeVita D. A., Lee T.-H., Murphy E. L. (2010). Human T cell leukemia virus type 1 infection drives spontaneous proliferation of natural killer cells. *Virulence*.

[29] Cooper M. A., Fehniger T. A., Caligiuri M. A. (2001). The biology of human natural killer-cell subsets. *Trends in Immunology*.

[30] Lewis G. K. (2014). Role of Fc-mediated antibody function in protective immunity against HIV-1. *Immunology*.

[31] Harrison D., Phillips J. H., Lanier L. L. (1991). Involvement of a metalloprotease in spontaneous and phorbol ester-induced release of natural killer cell-associated Fc gamma RIII (CD16-II). *The Journal of Immunology*.

[32] Jing Y., Ni Z., Wu J. (2015). Identification of an ADAM17 cleavage region in human CD16 (Fc*γ*RIII) and the engineering of a non-cleavable version of the receptor in NK cells. *PLoS One*.

[33] Wang Y., Wu J., Newton R., Bahaie N. S., Long C., Walcheck B. (2013). ADAM17 cleaves CD16b (Fc*γ*RIIIb) in human neutrophils. *Biochimica et Biophysica Acta (BBA) - Molecular Cell Research*.

[34] Azakami K., Sato T., Araya N. (2009). Severe loss of invariant NKT cells exhibiting anti–HTLV-1 activity in patients with HTLV-1–associated disorders. *Blood*.

[35] Ndhlovu L. C., Snyder-Cappione J. E., Carvalho K. I. (2009). Lower numbers of circulating natural killer T (NK T) cells in individuals with human T lymphotropic virus type 1 (HTLV-1) associated neurological disease. *Clinical & Experimental Immunology*.

[36] Gondois-Rey F., Chéret A., Granjeaud S. (2017). NKG2C^+^ memory-like NK cells contribute to the control of HIV viremia during primary infection: Optiprim-ANRS 147. *Clinical & Translational Immunology*.

[37] Cimini E., Viola D., Cabeza-Cabrerizo M. (2017). Different features of V*δ*2 T and NK cells in fatal and non-fatal human Ebola infections. *PLoS Neglected Tropical Diseases*.

[38] Spaan M., van Oord G. W., Janssen H. L. A., de Knegt R. J., Boonstra A. (2015). Longitudinal analysis of peripheral and intrahepatic NK cells in chronic HCV patients during antiviral therapy. *Antiviral Research*.

[39] Manel N., Battini J.-L., Taylor N., Sitbon M. (2005). HTLV-1 tropism and envelope receptor. *Oncogene*.

